# Toxoplasma Infection in an Immunocompetent Host: Possible Risk of Living with Multiple Cats

**DOI:** 10.7759/cureus.1103

**Published:** 2017-03-19

**Authors:** Halis Kaan Akturk, David Sotello, Allen Ameri, Ahmed S Abuzaid, Ana Marcella Rivas, Priyanka Vashisht

**Affiliations:** 1 Barbara Davis Center for Diabetes, University of Colorado-Anschutz Medical Campus; 2 Department of Infectious Disease, Mayo Clinic; 3 Infectious Disease, Creighton University; 4 Cardiology, Christiana Care Health System; 5 Endocrinology, Mayo Clinic; 6 Department of Rheumatology, University of Nebraska

**Keywords:** cats, toxoplasma gondii, brain abscess, immunocompetent, headache, parasites, multiple sclerosis, neurological symptoms, mri, ct

## Abstract

A 32-year-old man presented with agitation, headache, and confusion. He was immunocompetent and had been living with multiple cats for many years. His vital signs were stable. He was afebrile. Multiple blood tests did not show any serious problem. Brain magnetic resonance imaging (MRI) revealed multiple ring-enhancing white matter lesions. Cerebrospinal fluid analysis did not show any signs of infection. Based on a presumptive diagnosis of multiple sclerosis, high-dose corticosteroid treatment was started. However, this caused worsening of the symptoms and increased the size of the lesions. Corticosteroids were discontinued and biopsy was done. Biopsy of the lesions confirmed *Toxoplasma gondii* infection, and treatment with pyrimethamine/sulfadiazine was initiated. Treatment decreased the size of the lesions dramatically.

*Toxoplasma* infection of the central nervous system (CNS) is rare in immunocompetent hosts. Living with multiple cats is believed to be a risk factor for *Toxoplasma* infection in immunocompetent hosts.

## Introduction

Cognitive impairment in a young man is a challenging presentation that needs rapid evaluation. Toxoplasmosis is an infection caused by an intracellular protozoan parasite, Toxoplasma gondii [1]. The complete reproductive cycle occurs only in felines. There are various modes of transmission: ingestion of infectious oocysts from soil contaminated with cat feces, ingestion of tissue cysts from the meat of an infected animal, vertical transmission, blood transfusion, and organ transplant [1].

Toxoplasmosis is most commonly reported in immunocompromised states such as human immunodeficiency virus (HIV) infection, chemotherapy, and bone marrow transplant. There are a few case reports in immunocompetent patients [2-4]. We report a case of severe central nervous system (CNS) Toxoplasma infection in an immunocompetent host who lives with multiple cats.

## Case presentation

A previously healthy 32-year-old white man presented with gradually increasing agitation and confusion. His friends mentioned that he displayed personality changes recently and was more combative and forgetful. The patient was complaining of severe recurrent headaches that started a few months ago and were not accompanied by nausea or vomiting. He had a history of alcohol, tobacco, cocaine, and methamphetamine use for many years. There was no history of seizures, loss of consciousness, syncope, fever, rash, weakness, or involuntary movements. The patient also denied recent travel, camping, and contact with sick persons. There was no family history of neurologic disorders or CNS tumors. The patient was living alone with multiple cats for a long time (four cats for six years).

The patient was afebrile. Heart rate was 90 beats/min, and blood pressure was 115/75 mm Hg. Neurologic examination revealed no motor or sensory deficits. Cranial nerves examination was normal. Bilateral reflexes and gait were normal. However, slow comprehension, poor attention span, naming difficulties, and impaired short-term memory were noticed. Mini–Mental State Examination showed moderate cognitive impairment. The rest of the physical examination was unremarkable.

Laboratory investigation showed normal results of complete blood cell count and basic renal panel. Vitamin B12, folic acid, free thyroxine, thyroid-stimulating hormone, and C-reactive protein levels and erythrocyte sedimentation rate were also within normal limits. Urine drug screen was positive for cocaine.

**Figure 1:**
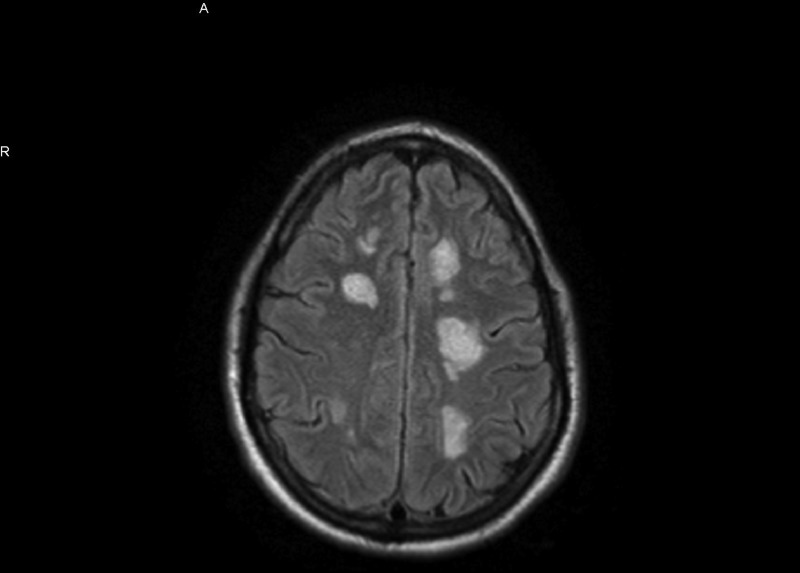
MRI with contrast shows ring-enhancing multiple bilateral lesions. Bilateral white lesions seen in MRI

**Figure 2:**
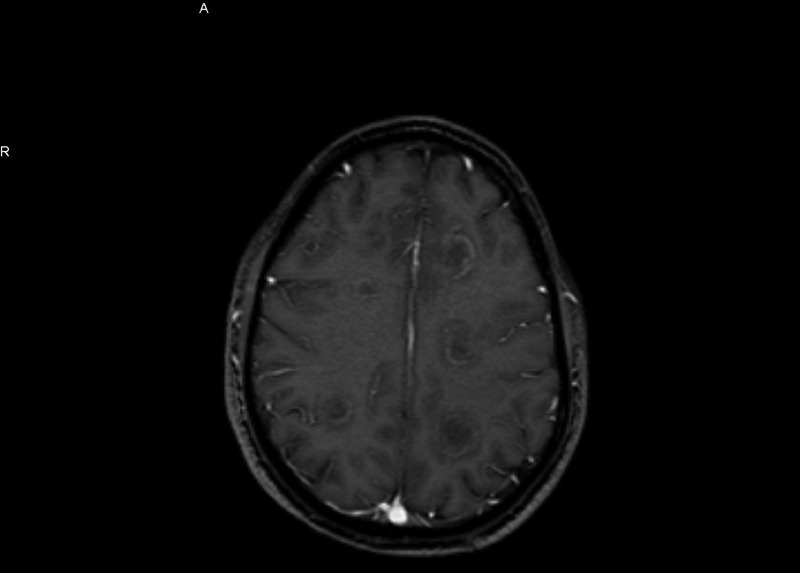
MRI without contrast shows ring-enhancing multiple bilateral lesions.

Computed tomography (CT) of the head showed multifocal white matter lesions. Magnetic resonance imaging (MRI) of the brain revealed extensive cortical and subcortical concentric ring-enhancing white matter lesions throughout both cerebral hemispheres (Figures 1-2).

 

**Figure 3:**
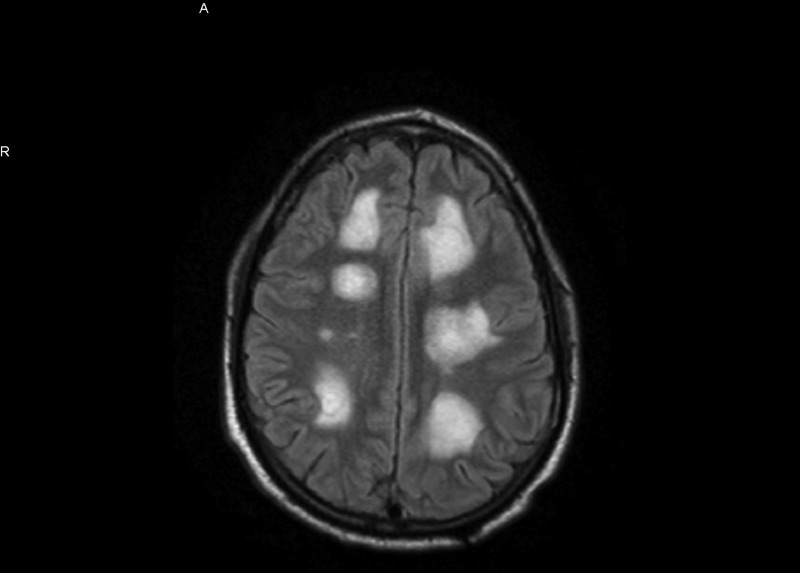
MRI with contrast after corticosteroid treatment shows increased size of the lesions. Bilateral white lesions seen in MRI

**Figure 4:**
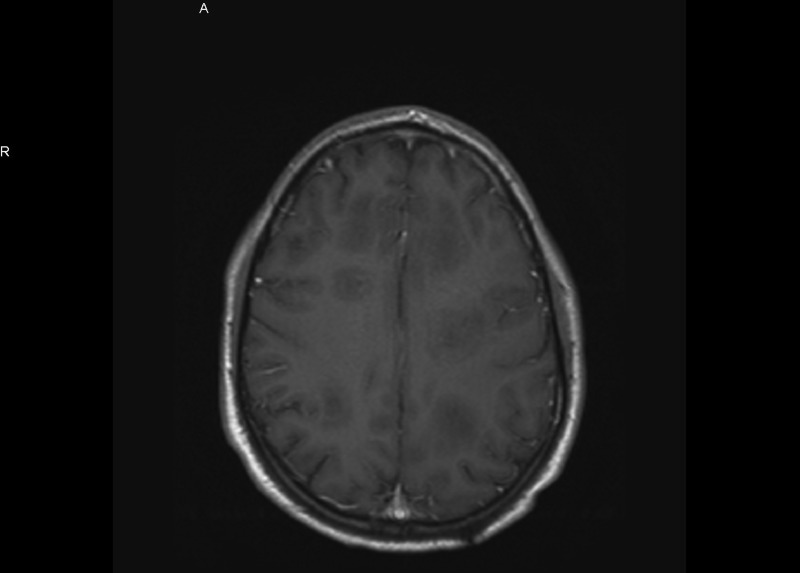
MRI without contrast after corticosteroid treatment shows increased size of the lesions.

The lesions were more prominent in left parietal and frontal lobes. The initial differential diagnosis was brain abscesses vs demyelinating disease. MRI of the spine showed no abnormalities. Cerebrospinal fluid (CSF) analysis showed elevated protein levels but no cells. Myelin basic protein and oligoclonal bands in the CSF were negative. Results of visual evoked potential studies were normal bilaterally. No vegetations were noted on the transthoracic echocardiogram. Ophthalmologic examination showed no evidence of chorioretinitis. Blood cultures and HIV test (4th generation enzyme-linked immunosorbent assay [ELISA]) were negative. HIV ribonucleic acid (RNA) was undetectable. CD4 count was 783 cells/mcl, Serum Toxoplasma Immunoglobulin M (IgM) was negative and Toxoplasma Immunoglobulin G (IgG) was positive (>12 IU/ml). Serum immunoglobulin levels were within normal limits. CSF serologic tests for cytomegalovirus, herpes, Toxoplasma, and JC/BK virus were negative. CT scan of the chest/abdomen/pelvis did not identify a primary malignancy. Based on a presumptive diagnosis of a demyelinating disorder (rare multiple sclerosis variant), the patient was given high-dose intravenous corticosteroids for seven days. Follow-up MRI showed increased number and size of brain lesions (Figures 3-4).

 

**Figure 5:**
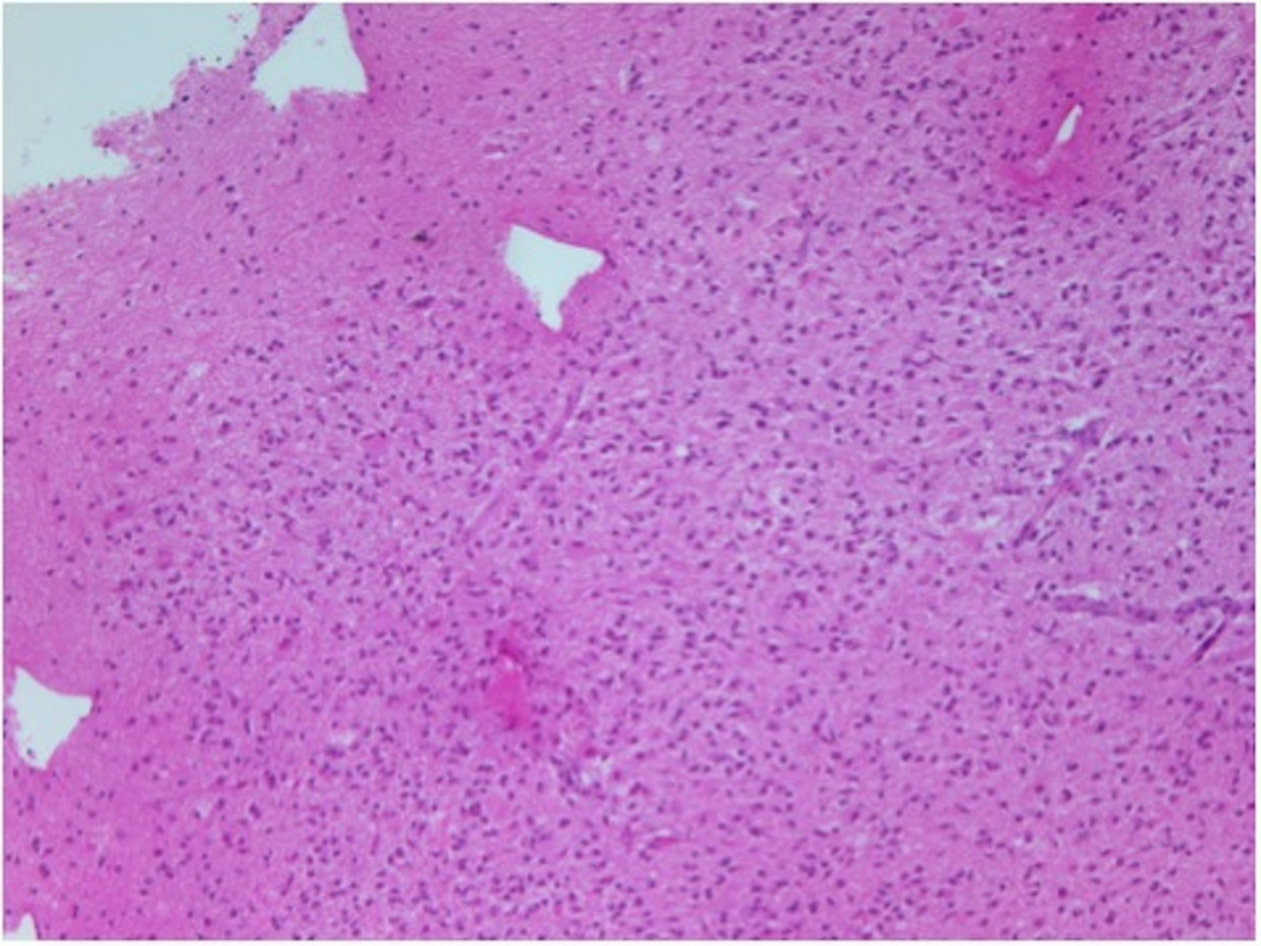
Biopsy shows increased number of inflammatory cells.

**Figure 6:**
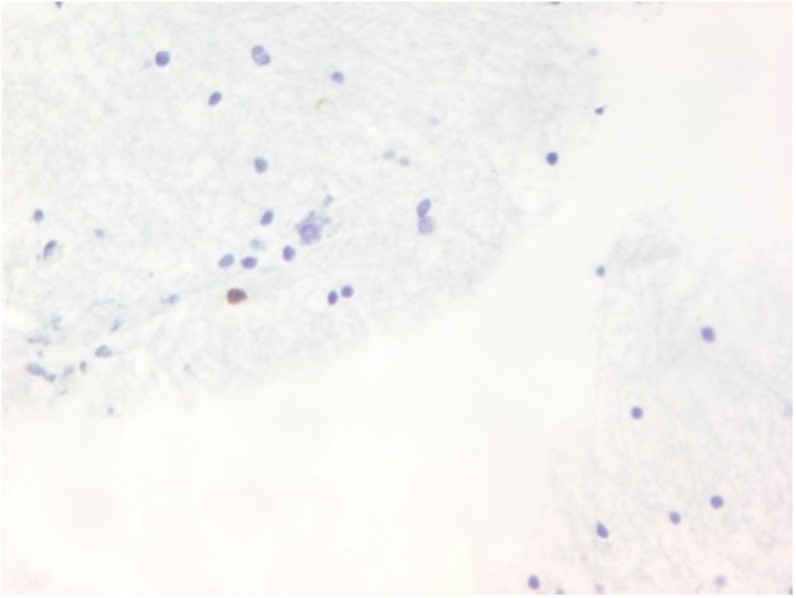
Biopsy shows basophilic parasites in cysts. Blue lesions across the specimen as seen in the picture

A stereotactic brain biopsy was done, and CNS toxoplasmosis was reported after biopsy and polymerase chain reaction analysis (Figures 5-6).

 

**Figure 7:**
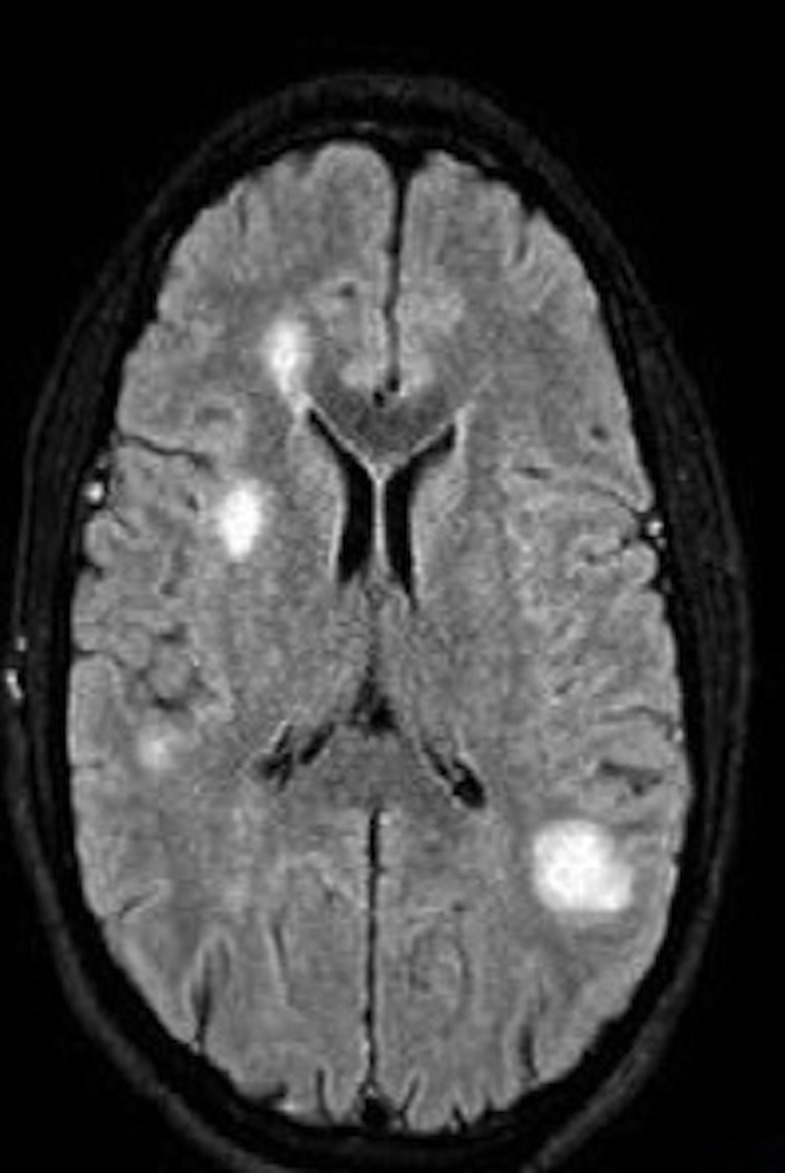
MRI after antimicrobial therapy shows decreased size of the lesions.

**Figure 8:**
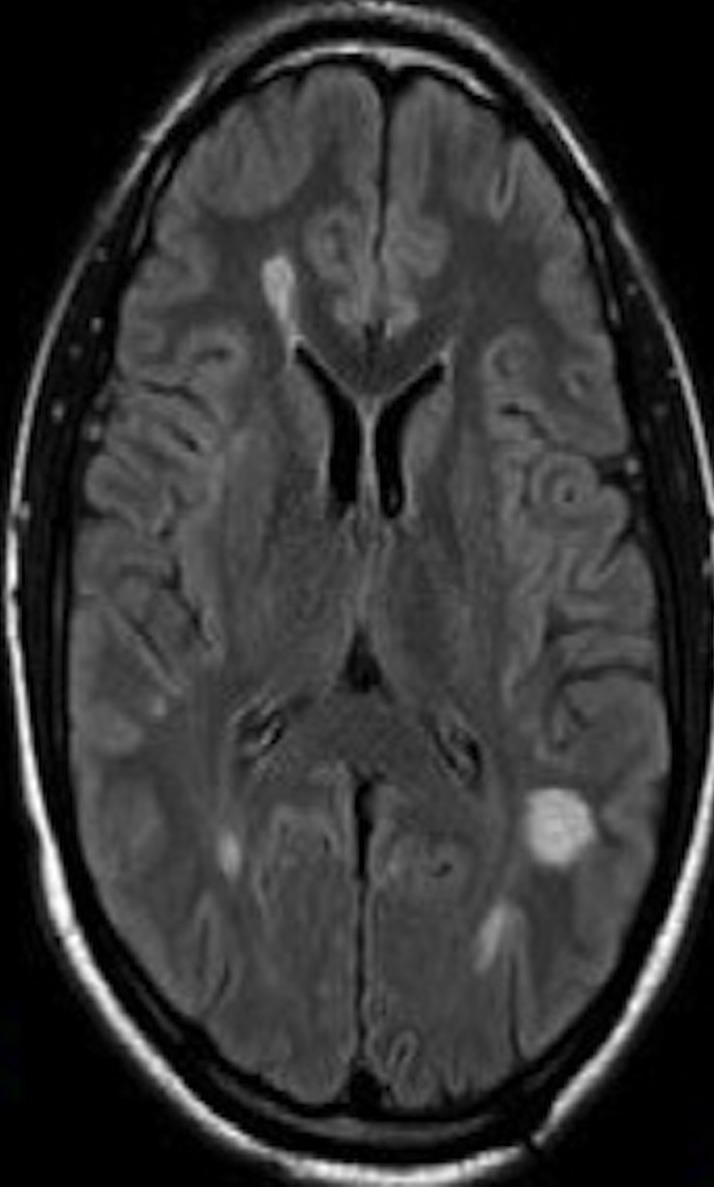
MRI after antimicrobial therapy shows decreased size of the lesions. Decreased white lesions seen in MRI

Pyrimethamine 200 mg loading dose, followed by 75 mg daily, sulfadiazine 1.5 g four times daily, and leucovorin 25 mg daily were started and continued for a total of eight weeks. The patient had significant clinical improvement associated with dramatic decrease in number and size of the lesions on follow-up brain imaging within one month (Figures 7-8)

 

The patient was followed up every three months with serial brain imaging. Dramatic clinical improvement was noticed in memory, concentration, and other neurologic findings, eventually returning to his normal baseline cognitive status.

## Discussion

This was a rare clinical presentation of widespread concentric white matter lesions in a young immunocompetent drug abuser with daily contact with cats. The differential diagnosis of ring-enhancing lesions is broad (Table 1).

**Table 1:** The differential diagnosis of ring-enhancing lesions in white matter

Multiple Sclerosis
Variant of multiple sclerosis: Balo concentric sclerosis
Brain abscesses: bacterial, Toxoplasma, cytomegalovirus, mycobacterial
Metastatic lesions
Central nervous system lymphoma
Deep white matter ischemia
Acute disseminated encephalomyelitis
Progressive multifocal leukoencephalopathy
Posterior reversible encephalopathy syndrome

At the initial presentation, there was a concern for demyelinating disorders. Absence of significant neurologic deficits, absence of markers for demyelinating disease and worsening symptoms and radiologic findings with high-dose corticosteroid therapy argue against a diagnosis of demyelinating disorder. Oligoclonal bands and CSF protein are nonspecific signs of multiple sclerosis and could signify inflammation. Improvement of the lesions with pyrimethamine/sulfadiazine makes CNS toxoplasmosis more likely.

Most commonly, Toxoplasma infection causes painless cervical lymphadenopathy [5]. The clinical manifestations of acquired toxoplasmosis in immunocompetent individuals have been reported to rarely include localized neurologic signs; in contrast, such signs are frequent in immunosuppressed patients [6-8].

Typically MRI reveals multiple lesions, commonly in the deep central nuclei and the posterior fossa at the gray-white matter junction with intense rim enhancement [9]. Histopathologic examination typically shows thick wall abscess and necrotic tissue with inflammatory cells. The sensitivity of immunostains is poor.

Toxoplasmosis generally does not need treatment in immunocompetent, nonpregnant patients unless their symptoms are severe or prolonged. In that case, the same medications are used as for immunosuppressed patients, but lower doses are sufficient. The usual duration of treatment is two to four weeks. In our case, we decided to treat at higher doses and for a longer period due to the severity of symptoms and radiologic findings. First-line regimens include pyrimethamine with sulfadiazine. Folinic acid supplements should be given to all patients receiving pyrimethamine. Alternative regimens include pyrimethamine combined with clindamycin azithromycin or atovaquone. Trimethoprim/sulfamethoxazole is an effective alternative treatment option.

## Conclusions

This case highlights the importance of considering infections of the CNS when investigating space-occupying lesions. The general conception is that CNS toxoplasmosis is seen in patients who have HIV or are otherwise immunocompromised. However, there are a few case reports involving patients who are not immunocompromised. A recent review showed a possible causal relationship between postnatal toxoplasmosis and epilepsy [10]. Biopsy with PCR is essential for the diagnosis. In our case, CSF antibody testing for Toxoplasma infection was negative but biopsy and PCR confirmed the diagnosis of toxoplasmosis. Evidence of a negative Toxoplasma IgM and a positive IgG implicates reactivation of Toxoplasmosis likely from prolonged exposure to felines. Exacerbation of symptoms and increasing size of the lesions after systemic corticosteroid therapy should increase the suspicion for an infectious cause. We believe that close contact with multiple cats increases the risk of CNS toxoplasmosis in immunocompetent individuals.
